# Evaluating the Reproducibility of Amplicon Sequencing Data Derived from Deep-Sea Cold Seep Sediment-Associated Microbiota

**DOI:** 10.1128/spectrum.04048-22

**Published:** 2023-04-19

**Authors:** Jie Kong, Jingchun Feng, Liwei Sun, Si Zhang

**Affiliations:** a Southern Marine Science and Engineering Guangdong Laboratory (Guangzhou), Guangdong, China; b Guangdong University of Technology, Guangzhou, China; University of Minnesota Twin Cities

**Keywords:** cold seep sediments, technical replicates, bacterial community, alpha diversity, beta-diversity, Illumina HiSeq platform

## Abstract

Benefiting from the rapid developments and wide applications of high-throughput sequencing, great advancements have been made in investigating microbiota, which are highly diverse and play key roles in both element cycling and the energy flow of ecosystems. There have been inherent limitations of amplicon sequencing that could introduce uncertainty and raise concerns about the accuracy and reproducibility of this technology. However, studies focusing on the reproducibility of amplicon sequencing are limited, especially in characterizing microbial communities in deep-sea sediments. To evaluate reproducibility, 118 deep-sea sediment samples were used for 16S rRNA gene sequencing in technical replicates (repeated measurements of the same sample) that demonstrate the variability of amplicon sequencing. The average occurrence-based overlaps were 35.98% and 27.02% between two and among three technical replicates, respectively, whereas their abundance-based overlaps reached 84.88% and 83.16%, respectively. Although variations of alpha and beta diversity indices were found between/among technical replicates, alpha diversity indices were similar across samples, and the average beta diversity indices were much smaller for technical replicates than among samples. Moreover, clustering methods (i.e., operational taxonomic units [OTUs] and amplicon sequence variants [ASVs]) were shown to have little impact on the alpha and beta diversity patterns of microbial communities. Taken together, although there are variations between/among technical replicates, amplicon sequencing is still a powerful tool with which to reveal diversity patterns of microbiota in deep-sea sediments.

**IMPORTANCE** The reproducibility of amplicon sequencing is vital for whether the diversities of microbial communities could be accurately estimated. Thus, reproducibility influences the drawing of sound ecological conclusions. Nevertheless, few studies have focused on the reproducibility of microbial communities that are characterized by amplicon sequencing, and studies focusing on microbiota in deep-sea sediments have been especially lacking. In this study, we evaluated the reproducibility of amplicon sequencing targeting microbiota in deep-sea sediments of cold seep. Our results revealed that there were variations between/among technical replicates and that amplicon sequencing was still a powerful tool with which to characterize the diversities of microbial communities in deep-sea sediments. This study provides valuable guidelines for the reproducibility evaluation of future work in experimental design and interpretation.

## INTRODUCTION

Microbiota are highly diverse and abundant, and they play key roles in element cycling and in the flow of energy in ecosystems (1, 2). Because of the negative influences of ongoing global climate change and human activities, it is unprecedentedly important to comprehensively characterize microbial communities (3, 4). Although great improvements have been made in microbial cultivation, the majority of microbiota are still hitherto unculturable, which hampers the detailed characterization of microbial communities (5). Scientists have benefited from the rapid development of culture-independent approaches, especially high-throughput sequencing (HTS), to comprehensively document microbiota from a variety of ecosystems (1, 6–9).

The HTS is one of the most well-developed technologies. Recent rapid developments and the wide applications of the HTS of marker genes have greatly advanced research concerning community structure and composition, especially studies targeting microbial communities (1). However, there have been inherent limitations of amplicon sequencing, and these might introduce an accumulation of uncertainty in almost every decision-making step, including sampling, DNA extraction, polymerase chain reaction [PCR] amplification, sequencing, and sequence data processing (10, 11). Many studies have been conducted to assess this possible uncertainty and to provide recommendations for better practices (10–15). The reproducibility of amplicon sequencing is vital for whether the diversities of microbial communities can be accurately estimated. Thus, reproducibility influences the drawing of sound ecological conclusions. Nevertheless, few studies have focused on the reproducibility of microbial communities characterized by amplicon sequencing (16–21), and studies focusing on microbiota in deep-sea sediments have been especially lacking (22).

Deep-sea sediment is one of the largest ecosystems on Earth, harboring diverse and abundant biomes among which microbiota are dominant and regulate the biogeochemical cycle (23, 24). Cold seeps, largely and widely discovered in continental margins across the world ocean, are characterized by a number of biogeochemical reactions and support one of the most diverse biomes on the seabed (25). The Haima cold seep is an active cold seep that was discovered on the northwestern slope of the South China Sea in 2015 (26). Since then, the Haima cold seep has been chosen as an excellent ecosystem for multidisciplinary research, including the characterization of microbial communities via marker gene sequencing (27). The Illumina HiSeq has become a dominant platform for the sequencing of marker genes of microbial communities, mainly due to its high throughput and high accuracy, together with its low cost (6), which may help mitigate the uncertainty of technical reproducibility for this technology. In this study, deep-sea sediments were collected from the Haima cold seep at various depths, ranging from the surface to 800 cm below the seafloor. Technical replicates of 118 samples were used to evaluate the reproducibility of amplicon sequencing (Illumina HiSeq sequencing), using the same processes from DNA extraction to bioinformatic analyses. Our main goals were to answer the following questions: (i) what is the extent of technical replicate variation when amplicon sequencing is used to target microbial communities in deep-sea sediments, and (ii) do technical reproducibility and the choice of sequence clustering method influence the evaluation of microbial diversities.

## RESULTS

### Occurrence-based and abundance-based overlaps between or among technical replicates.

Without rarefaction, the sequence number of the 344 samples ranged from 57,049 to 68,078 with a mean value of 65,895 (standard deviation [SD] equal to 1,295) for the operational taxonomic unit (OTU)-based pipeline. The sequence numbers ranged from 59,125 to 68,637 (mean ± SD, 67,436 ± 1,046) for the ASV-based pipeline (Tables S2 and S4). The OTU number varied from 57 to 2,420 (mean ± SD, 372.7 ± 428) when the sequences were not rarefied. When rarefaction was applied, the OTU number ranged from 51 to 2,061 (mean ± SD, 349.8 ± 393) (Tables S3 and S4).

**FIG 1:**
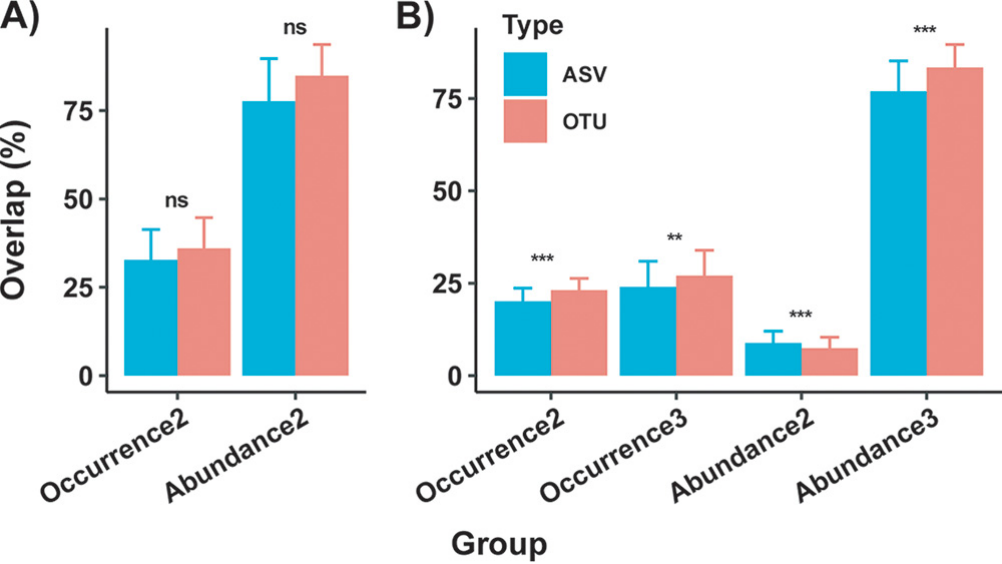
The OTU-based and ASV-based overlaps between two (A) and among three (B) technical replicates. The bar and error bar show the mean and standard deviation values of the overlaps, respectively. The comparisons between OTU-based and ASV-based mean overlaps were tested using a one-way analysis of variance and Tukey’s honestly significant differences [HSD] test. ns, *P* value >0.05; **, 0.001 < *P* < 0.01; ***, *P* < 0.001. Occurrence, only the occurrence (presence or absence) of OTUs/ASVs was considered; abundance, the sequence abundances of OTUs/ASVs were considered. 2 and 3 represent overlaps shared by two and three technical replicates, respectively. For each sample, a value of the OTU-based/ASV-based overlap between two or among three technical replicates was calculated.

The average occurrence-based OTU overlap was 35.98 ± 8.75% (mean ± standard deviation) for two technical replicates (Fig. 1A; Table S5). For three technical replicates, the average occurrence-based OTU overlap shared by three replicates was 27.02 ± 6.9%, together with 23.13 + 3.19% OTUs shared by two replicates (Fig. 1B; Table S5). When sequence abundances were taken into consideration, the overlaps between/among replicates were much higher than were the occurrence-based overlaps. The average abundance-based overlap was 84.88 ± 8.80% for two technical replicates, whereas for three technical replicates, the sequence abundances shared by three replicates accounted for 83.16 ± 6.44%, with 7.31 ± 3.11% sequences shared by two replicates (Fig. 1; Table S5). Compared to the OTU-based overlaps, the average ASV-based overlaps were significantly lower for both occurrence-based and abundance-based overlaps shared by three technical replicates (Fig. 1B; Table S5).

### Variability of alpha and beta diversity indices between/among technical replicates.

The OTU-based percent relative ranges (PRRs) of the Richness index varied from 3.1% to 97.8% (mean value, 43.2%) and from 0.6% to 177.8% (mean value, 54.7%) for two and three technical replicates, respectively (Table 1). The average OTU-based PRRs of the Shannon index were 22.2% and 16.0% for two and three technical replicates, respectively, and these values were much lower than those of the richness index (Table 1). As for the ASV-based PRRs, the average PRRs of the richness index (two replicates, 41.5%; three replicates, 54.0%) were much higher than those of the Shannon index (two replicates, 16.0%; three replicates, 22.7%), which was similar to the patterns of the OTU-based PRRs (Table 1).

**TABLE 1:** Statistical summary of the values of the percent relative range (%) for the alpha indices among the technical replicates^*a*^

Statistic	Two technical replicates				Three technical replicates			
	OTU-based		ASV-based		OTU-based		ASV-based	
	Richness	Shannon	Richness	Shannon	Richness	Shannon	Richness	Shannon
Min	3.1	0.6	2.2	0.4	0.6	0.7	4.9	1.2
Mean	43.2	22.2	41.5	16.0	54.7	33.8	54.0	22.7
Max	97.8	45.7	86.6	34.1	177.8	131.9	193.4	80.9
SD	32.9	15.7	29.9	11.7	38.9	23.8	40.7	16.6

a The percent relative range (PRR) represents the percentage ratio of the range to the average value. A larger PRR value indicates more variability between two or among three technical replicates. The headers of “Two technical replicates” and “Three technical replicates” represent data sets with two (10 samples) and three (108 samples) technical replicates. respectively. The headers of OTU-based and ASV-based indicate the sequence clustering method of the data set. The four data sets were rarefied as mentioned in Materials and Methods. Min, minimum; Max, maximum; SD, standard deviation.

For the beta diversity, the average dissimilarity indices based on occurrence were slightly but not significantly (*P* > 0.05) larger than those based on sequence abundances for both two and three technical replicates (Table 2). For the OTU-based beta diversity, the average occurrence-based and abundance-based dissimilarities between two technical replicates were 0.640 and 0.625, respectively, whereas for three technical replicates, the average dissimilarities based on occurrence and abundance were 0.612 and 0.594, with their average PRRs being equal to 11.6% and 11.1%, respectively (Table 2). Compared to the OTU-based beta diversity values, the ASV-based average dissimilarities were larger between/among technical replicates (Table 2).

**TABLE 2:** Statistical summary of the beta diversity indices (Bray-Curtis dissimilarities) among the technical replicates^*a*^

Statistic	Two technical replicates				Three technical replicates			
	OTU-based		ASV-based		OTU-based		ASV-based	
	O-based	A-based	O-based	A-based	O-based	A-based	O-based	A-based
Min	0.528	0.520	0.548	0.489	0.387	0.345	0.373	0.377
Mean	0.640	0.625	0.673	0.665	0.612	0.594	0.657	0.630
Max	0.814	0.812	0.830	0.810	0.756	0.729	0.804	0.774
PRR.m					11.6%	11.1%	9.6%	9.7%
All.m	0.816	0.810	0.836	0.823	0.779	0.765	0.813	0.786

a The numbers of sequences were log1p() transformed before a pairwise dissimilarity calculation. The Bray-Curtis dissimilarity indices were calculated using the vegdist function in the R package vegan. The four data sets were rarefied as mentioned in Materials and Methods. O-based, occurrence-based dissimilarity; A-based, abundance-based dissimilarity; PRR.m, mean percent relative range of the technical replicates; All.m, mean pairwise dissimilarity for all samples.

### Robustness of amplicon sequencing in diversity estimation.

All *P* values of Spearman correlation tests were <0.05, indicating that technical replicates, though differing in alpha diversities for each sample, showed similar trends for both the richness and Shannon indices across samples. For two technical replicates, the average Spearman’s ρ values were 0.755 and 0.825 for the OTU-based richness and Shannon indices, respectively (Table 3; Fig. S2). As for three technical replicates, the average ρ values were 0.797 and 0.733 for the OTU-based richness and Shannon indices, respectively (Table 3; Fig. S2).

**TABLE 3:** Statistical summary of the Spearman’s ρ values for the alpha indices among the technical replicates^*a*^

Statistic	Two technical replicates				Three technical replicates			
	OTU		ASV		OTU		ASV	
	Richness	Shannon	Richness	Shannon	Richness	Shannon	Richness	Shannon
Min	0.685	0.733	0.721	0.733	0.714	0.645	0.728	0.663
Mean	0.755	0.825	0.779	0.797	0.797	0.733	0.803	0.744
Max	0.830	0.915	0.867	0.879	0.879	0.826	0.883	0.847
SD	0.035	0.038	0.036	0.037	0.025	0.031	0.024	0.030

a The four data sets were rarefied as mentioned in Materials and Methods. For each data set, the values of the technical replicates from the same sample were randomly divided into two groups, with each group containing one technical replicate. Then, the Spearman correlation coefficient was calculated between those two groups. The random division and Spearman correlation test were repeated 999 times, which resulted in 999 ρ values for each data set. All of the *P* values of the Spearman correlation tests were <0.05.

The average dissimilarities calculated from all samples with technical replicates were much larger than those calculated from technical replicates of each sample for both OTU-based/ASV-based and occurrence-based/abundance-based data sets (Table 2). Further analysis revealed that the dissimilarities between technical replicates were significantly lower than were the intersample dissimilarities (*P* < 0.001).

### Assessing the agreement of sequence clustering on diversity estimation.

**FIG 2:**
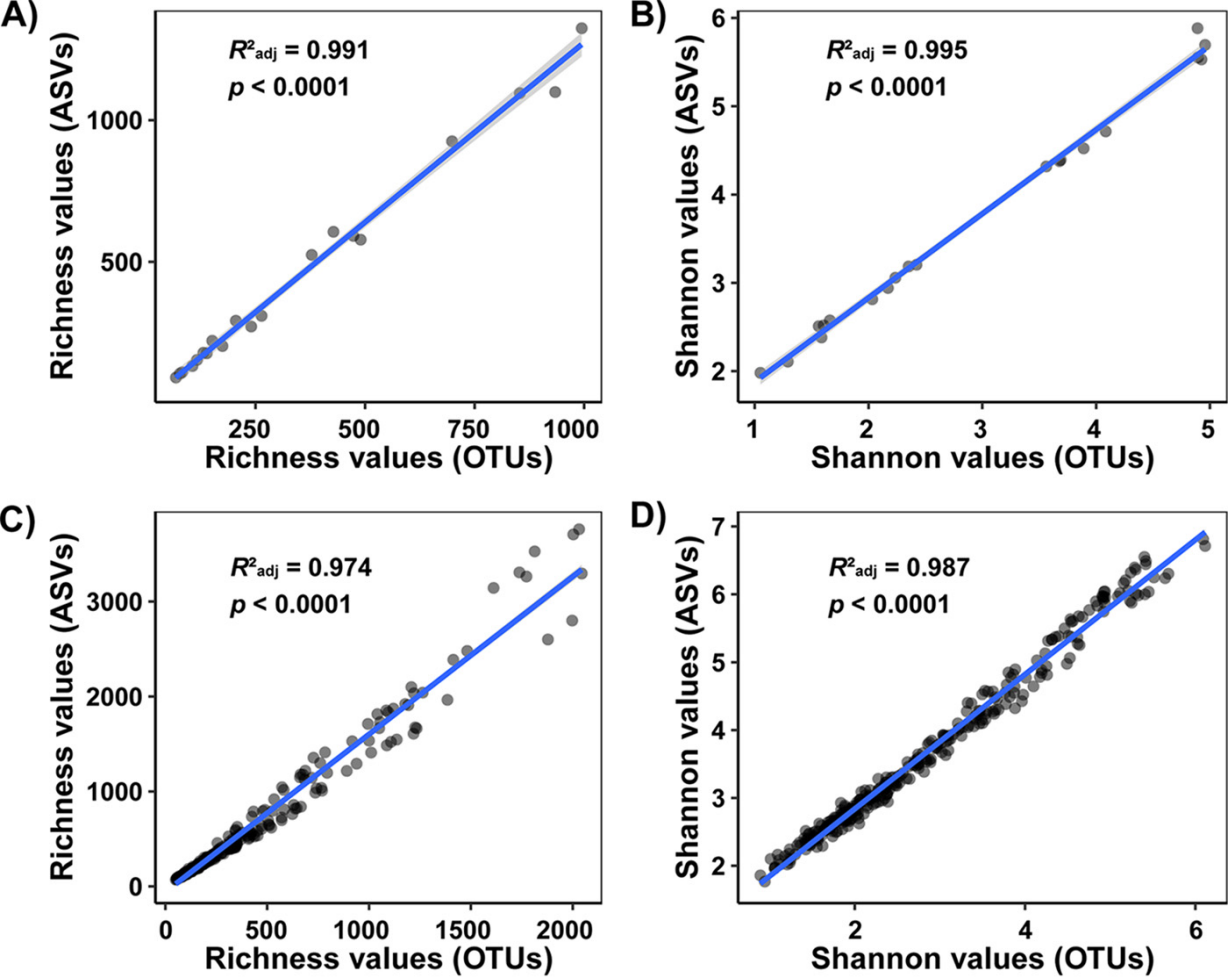
Linear regressions showing the relationships between OTU-based and ASV-based alpha diversity indices. (A) Richness indices of the 10 samples with 2 technical replicates. (B) Shannon indices of the 10 samples with 2 technical replicates. (C) Richness indices of the 108 samples with 3 technical replicates. (D) Shannon indices of the 108 samples with 3 technical replicates.

**FIG 3:**
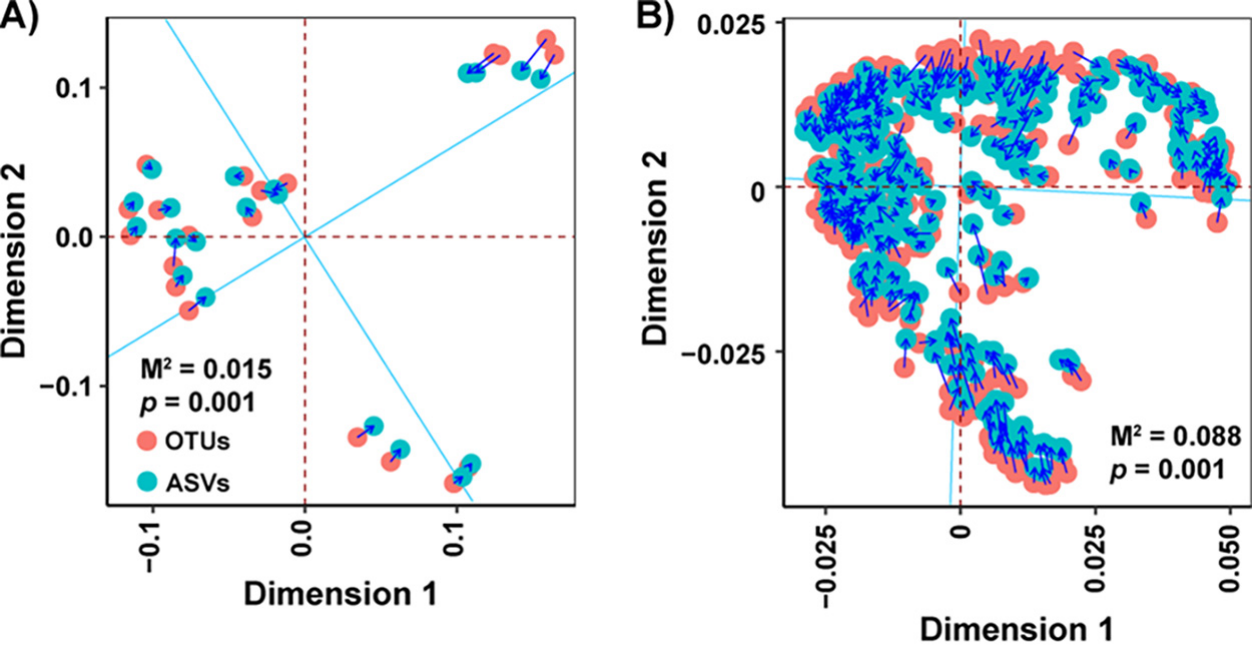
Procrustes analysis to evaluate the agreement regarding the distribution patterns of the OTU-based and ASV-based bacterial communities. (A) The 10 samples with 2 technical replicates. (B) The 108 samples with 3 technical replicates. A principal coordinates analysis based on the Hellinger-transformed data were applied, with statistical significance being measured via a Monte Carlo test and the M^2^ and *P* values indicating the goodness-of-fit and the significance of the Monte Carlo test, respectively. Blue lines between pairs of points indicate that the two connected points represent the same sample.

As revealed above, clustering methods (i.e., OTUs and ASVs) were shown to have impacts on estimating the exact values of the alpha and beta diversities of microbial communities in deep-sea sediment when technical replicates were taken into consideration. However, linear regression analyses revealed strong linear correlations between OTU-based and ASV-based alpha diversity indices with an adjusted *R*^2^ value of >0.97 (Fig. 2), indicating that OTU-based and ASV-based sequence clustering methods presented similar alpha diversity patterns. Moreover, a Procrustes analysis showed remarkable agreement between OTU-based and ASV-based distribution patterns for both two (M^2^ = 0.015; *P* = 0.001) and three (M^2^ = 0.088; *P* = 0.001) technical replicates (Fig. 3). Collectively, these results showed that OTU-based and ASV-based clustering methods had little impact on the alpha and beta diversity patterns of microbial communities in deep-sea sediments.

## DISCUSSION

The technical reproducibility of amplicon sequencing is a critical issue for HTS-based studies, not only because it is vital for whether the diversities of microbial communities can be accurately and stably estimated but also because it determines whether and how this technology should be used in a specific scenario. Though HTS-based amplicon sequencing has been applied in numerous studies, few studies have focused on the reproducibility of this technology, with results being especially lacking for the targeting of microbial communities in deep-sea sediments (17–22). Because of the complexity of natural microbial communities, together with the imperfections of all available technologies, it is impossible to quantify the differences between the observed and the actual microbial communities. In this study, we assessed the technical reproducibility of amplicon sequencing using Illumina HiSeq data from 118 deep-sea sediments with technical replicates.

### Variability of technical replicates for amplicon sequencing.

Based on our results, the occurrence-based overlaps were 35.98% and 27.02% between two and among three technical replicates when an Illumina HiSeq platform was applied to target microbiota in deep-sea sediments (Fig. 1; Table S5). The average occurrence-based OTU overlaps were observed to be 17.2% and 8.2% between two and among three technical replicates, respectively, with 454 pyrosequencing soil microbial communities (21), which are much lower than our results. Wen et al. (2017) attributed this to technical differences, as Illumina sequencing, compared to 454 pyrosequencing, has lower sequencing error rates, which result in lower percentages of spurious OTUs (19). However, the OTU overlaps between two technical replicates have been shown to be 39.8% and 36.4% for plankton communities from freshwater and marine harbors, respectively, as revealed by 454 pyrosequencing (20). Obviously, technical differences (454 pyrosequencing versus Illumina sequencing) alone could not explain the observed differences of overlap between/among technical replicates. Moreover, when Illumina MiSeq sequencing was used to evaluate the technical reproducibility of soil microbial investigation, the average OTU overlaps have made up 33.4% and 20.2% of the total OTU numbers between two and among three technical replicates, respectively (19), which is in concordance with our results.

Additionally, the abundance-based overlaps, relative to the occurrence-based overlaps, were much higher both between two and among three technical replicates (Fig. 1), indicating that a large number of OTUs that were not shared by all replicates made up a small portion of the sequence abundances. Similarly, the abundance-based overlaps, compared to the occurrence-based overlaps, have been observed to be much higher for two (85.6%) and three (81.2%) replicates (19). It would be reasonable to expect that overlaps between/among technical replicates increase with a higher threshold of minimum OTU/ASV sequence numbers being kept. In a recent work, technical replicates generated in two laboratories shared an average of 78.5% of all bacterial ASVs after filtering ASVs with sequences <100 (17). In this study, when the ASVs/OTUs with sequences <100 were filtered, the average ASV-based and OTU-based overlaps increased for both occurrence-based and abundance-based overlaps of two technical replicates as well as for abundance-based overlaps shared by three technical replicates (Tables S5 and S7). It should be noted that the majority of microbial OTUs/ASVs are usually in low abundance. Therefore, a high filtration threshold would result in the underestimation of low-abundant OTUs/ASVs, which might play important roles in community assembly and ecosystem functioning (28).

Alpha and beta diversity indices showed variations between/among technical replicates (Tables 1 and 2; Table S5; Fig. 1). It was noteworthy that, taking the richness index as example, the OTU-based PRR could reach as high as 177.8% and also as low as 0.6% (Table 1), indicating the importance of including technical replicates and taking the reproducibility of amplicon sequencing into consideration in future studies. It has been widely accepted that almost every decision-making step, including sampling, DNA extraction, PCR amplification, sequencing, and sequence data processing could introduce uncertainty into amplicon sequencing, which might result in skewed microbial communities, relative to the actual ones (10, 11). Comparing six bioinformatics pipelines, a recent study has revealed that there are pipeline-dependent and parameter-dependent biases for analyzing 16S rRNA amplicon sequencing data (29). Specifically, sequence rarefaction, one optional process of sequence processing, has been an issue of debate as a best practice (30–32). Samples with various sequence counts would introduce biases because of the fact that more OTUs/ASVs would be recovered for larger library sizes. The avoidance of rarefaction has also been advocated due to its random omission of valid data (31). Oppositely, repeated rarefying has been proposed as a tool with which to normalize library sizes (30). The conservation of larger library sizes has been observed to allow for the detection of more diversity, with minimal variation being observed between the iterations of rarefaction (30). To assess the effect of one-time rarefaction on diversity estimation in this study, repeated rarefaction (100 times) was applied to generate a representative set of data. This revealed that the rarefaction curves were almost saturated at the rarefaction depths used in this study (Fig. S4), suggesting that the current sequencing and rarefying depths were large enough to capture the community diversities for almost all samples, with limited variation between the iterations of rarefaction. To evaluate the effect of rarefaction on estimating the alpha diversity indices of technical replicates, we further calculated the PRR of the richness and Shannon indices, estimated both with and without rarefaction (Table S6). The PRR of OTU-based richness with and without rarefaction ranged from 0 to 22.3% (mean value, 5.6%), whereas the PRR of the OTU-based Shannon index varied from 0 to 1.8% (mean value, 0.3%). Compared to the PRRs of the alpha diversity indices between/among technical replicates (Table 1), the variability of the alpha diversity due to rarefaction was much lower, indicating that other processes are also contributing to the observed variability of technical replicates.

Beside the uncertainty introduced by all processes of amplicon sequencing (10, 11), the spatial heterogeneities of the microbial communities and their surrounding environmental conditions might be important factors that are contributing to the observed variability values of the alpha and beta diversities between/among technical replicates, as a tiny amount of sediment, as small as 0.2 g, was used in the DNA extraction for each technical replicate. Variations of microbial community structures at fine vertical scales (2 cm interval) have been observed for cold seep sediments (33), indicating the existence of microbial community heterogeneity at small scales of cold seep sediments. Hence, the observed variations of microbial communities between/among technical replicates might reflect the true community structure heterogeneity. Moreover, the effect of extracellular DNA presented in the sediments could not be excluded (34), as this effect that might distort the diversity estimates by significantly influencing the rare OTUs/ASVs. It should be noted that pooling several DNA samples that were extracted from the same sample is an alternative option of technical replicates. The effect of technical replicates and pooled DNA extractions on species detection has been evaluated, and whether or not to pool has been suggested to depend on the research question (35). Considering the variability of the technical reproducibility of amplicon sequencing, technical replicates should be recommended in future studies with caution, as there might be microbial structure heterogeneity at small scales, and this heterogeneity should be kept in mind when this technique is applied to target microbiota in deep-sea sediments.

### Robustness of amplicon sequencing on diversity estimation.

Although variations were found for overlaps, when considering the alpha and beta diversity indices between/among technical replicates (Tables 1 and 2; Table S5; Fig. 1), the alpha diversity indices presented similar patterns across samples (Table 3; Fig. S2), whereas the average beta diversity indices were much smaller between/among technical replicates than among samples (Table 2), indicating that diversity patterns were not significantly affected by the technical replicate variations. Similarly, low technical reproducibility has been found for pyrosequencing and Illumina MiSeq sequencing, but clear composition and structure differences have also been revealed for soil microbial communities between experimental groups (19, 21). Moreover, technical replicates have shown highly congruent measurements of alpha and beta diversities, together with taxonomic compositions of benthic bacterial communities in coastal sediments, and they have also revealed less variation than have biological replicates (17). Taken together, a conclusion could be made that amplicon sequencing is sufficient for analyzing microbial community turnover if the variations of technical replicates are much less than the targeted microbial turnover rate.

Our results indicated that clustering methods, such as OTUs and ASVs, have little impact on the alpha and beta diversity patterns of microbial communities in deep-sea sediments (Fig. 2 and 3). Thus, OTU-based and ASV-based analyses would present similar diversity patterns. Our results suggest that future work should focus on other aspects, rather than clustering methods, in influencing the technical reproducibility of amplicon sequencing. Although artificially defined OTUs clustered at a fixed similarity threshold have been customarily used and will continue to be used in many future studies, the replacement of OTUs by ASVs should be encouraged, as ASVs, relative to OTUs, are thought to provide finer taxonomic resolution and more reusable, reproducible, and comprehensive results (36). The fact that many factors might contribute to variations between/among technical replicates, including the heterogeneity of samples and the uncertainty of amplicon sequencing, appeals to standard methods and more studies for best practices. Given this fact, mock communities with known taxonomic affiliations are particularly recommended for use in working toward standards and best practices for future studies. Future work should focus on solving knowledge gaps, such as which factors are the key determinants influencing the reproducibility of amplicon sequencing and how to achieve better reproducibility of amplicon sequencing, which would greatly enhance the accuracy and applicability of amplicon sequencing.

### Conclusions.

In conclusion, our results revealed that there were variations between/among technical replicates when amplicon sequencing was used to characterize the diversities of microbial communities in deep-sea sediments. This emphasizes the importance of containing technical replicates and taking the uncertainty of amplicon sequencing into consideration for study design and the interpretation of results. Furthermore, our results showed that amplicon sequencing is a powerful tool with which to reveal the diversity patterns of microbiota in deep-sea sediments. It should be noted that the reproducibility of technical replicates could vary significantly among different samples. Many factors could contribute to the observed variations between/among technical replicates, including the heterogeneity of environmental variables and microbial communities as well as the uncertainty of amplicon sequencing that could be introduced by almost every decision-making step. The key determinants that influence the reproducibility of amplicon sequencing and whether they vary among different ecosystems remain unclear and are in need of further study.

## MATERIALS AND METHODS

### Collection and processing of deep-sea sediments.

To capture diverse microbial communities, five sediment cores were collected from five stations (seawater depths ranging from 1,366 to 1,483 m) located in the Haima cold seep in the northern South China Sea on May 20 and 21 of 2021 (Fig. S1). At each station, a sediment core longer than 600 cm was collected using a gravity corer. Once transported onto deck, subsamples of sediments for DNA extraction at a specific depth (relative to the top of the sediment core) (Table S1) were collected, vortexed, shaken, and stored at −80°C until further analysis. To avoid possible contaminations to our samples, several processes were applied in the field and in the laboratory. For example, only the inner part of sediment core was sampled for DNA extraction, and this was done using steel spoons that had been autoclaved and kept in 75% ethanol. Additionally, sterile tubes were used to store the sediment samples, and sterile latex gloves and surgical face masks were used during the DNA sample collection.

### DNA extraction, PCR, and sequencing.

For each sample, total genomic DNA extraction was conducted in technical replicates. For each technical replicate, about 200 mg of sediment were used to extract the total genomic DNA after vortex mixing. A NucleoSpin Soil Kit (Macherey-Nagel, Germany) was used to extract the total genomic DNA, following the manufacturer’s instructions. Gel electrophoresis (1% agarose gel) and a Qubit 4.0 Fluorometer with a Qubit 1×dsDNA HS Assay Kit (Thermo Fisher Scientific) were applied to evaluate the DNA quality, purity, and quantity. The primer set 338F (5′-ACTCCTACGGGAGGCAGCAG-3′) and 806R (5′-GGACTACHVGGGTWTCTAAT-3′), tagged with Illumina adapter, pad, and linker sequences, was used to target the V3 and V4 variable regions of the bacterial 16S rRNA gene (37). PCR was performed in a 50 μL reaction, including 30 ng DNA template, primer, and PCR master mix. PCR cycling started with 94°C for 3 min, and this was followed by 30 cycles of 94°C for 30 s, 56°C for 45 s, and 72°C for 45 s, with the process ending with a final extension at 72°C for 10 min. The PCR products were qualified via gel electrophoresis (1% agarose gel) and purified using Agencourt AMPure XP beads (Beckman Coulter, USA). This was followed by library construction, following the manufacturer’s instructions. The quality of the libraries was evaluated using an Agilent 2100 bioanalyzer (Agilent, USA). The validated libraries were sequenced on an Illumina HiSeq 2500 PE300 platform, which generated 2 × 300 bp paired-end reads, following the standard pipelines of Illumina. The DNA extraction, PCR, and 16S rRNA gene sequencing were conducted at BGI Genomics (Shenzhen, China).

### Processing of sequencing data.

Raw reads were processed by BGI Genomics to generate high quality sequences, including the removal of adapters, barcodes, primers, and low-quality sequences. The following steps for sequence processing and analyzing were conducted mainly with VSEARCH v2.7.0 (38, 39). The paired-end reads were merged, and this was followed by the discarding of sequences with low quality (total expected errors >1) or shorter than 300 bp (38). To assess whether clustering methods influenced the reproducibility of technical replicates, the customarily used OTUs (sequences clustered at a fixed similarity threshold) at a 97% similarity level as well as the recently developed ASVs (using exact sequence variants) were constructed using the commands cluster_fast (minsize = 2) and cluster_unoise (minsize = 8), respectively. Then, the command uchime_ref, based on the SILVA 132 database (40), and the command uchime3_denovo were applied for OTU-based and ASV-based chimera detection, respectively. The taxonomy assignments for the representative sequences of the OTUs/ASVs were BLAST-ed against SILVA 132 (40), using assign_taxonomy.py in QIIIME v.1.9.0 (41). Only OTUs/ASVs that were assigned to bacteria and archaea were kept, and this was followed by randomly rarefying sequences of each sample to the minimum sequence number in the data set.

In total, 118 sediment samples were successfully sequenced in technical replicates, including 10 samples with 2 technical replicates and 108 samples with 3 technical replicates. The following 4 data sets were constructed: (i) OTU-based data set for the 10 samples with 2 technical replicates (20 samples); (ii) ASV-based data set for the 10 samples with 2 technical replicates (20 samples); (iii) OTU-based data set for the 108 samples with 3 technical replicates (324 samples); (iv) ASV-based data set for the 108 samples with 3 technical replicates (324 samples). For each data set, random rarefaction was conducted. Thus, the sequence numbers of the samples in the aforementioned 4 data sets were rarefied to 57,047, 66,383, 59,001, and 59,021, respectively. To achieve rarefaction, the command alpha_rarefaction.py in QIIIME v.1.9.0 (41) was applied for each data set.

### Statistical analyses.

The observed OTU/ASV number (here, richness) and the Shannon indices, which are widely used in microbial community studies, were chosen as the representatives of alpha diversity. The Shannon index was calculated using the diversity function in the R package vegan 2.6-4 (42).

The occurrence-based (considering the presence or absence of OTUs/ASVs) and abundance-based (accounting for the sequence abundances) overlaps were calculated between/among technical replicates following (19) and using and the formula below:

$$P_{\mathrm{overlap}}=\frac{n\times N_{\mathrm{shared}}}{N_{\mathrm{total}}}\times 100\%.$$

The P_overlap_ (%) is the occurrence-based or abundance-based overlap between two or among three technical replicates. The *n* value, which is equal to either 2 or 3, represents the number of technical replicates. The N_shared_ value represents the OTUs (or ASVs) or sequence number shared by the *n* technical replicates. The N_total_ value represents the total OTUs (or ASVs) or sequence number of the *n* technical replicates.

The percent relative range (the percentage ratio of the range to the average value; PRR) of the alpha diversity indices was calculated to assess their variability between/among technical replicates. A larger PRR value means that more variability was observed between/among technical replicates. Both occurrence-based and abundance-based Bray-Curtis dissimilarities were calculated to estimate the beta diversity between/among technical replicates, using the vegdist function in the R package vegan 2.6-4 (42).

To assess the robustness of the alpha diversity indices between/among technical replicates, the values of the technical replicates from the same sample were randomly divided into two groups, with each containing one technical replicate. Then, the Spearman correlation coefficient was calculated between those two groups. The random division and Spearman correlation test were repeated 999 times, and a *P* value of <0.05 was considered to be indicative of a statistically significant result. The significant Spearman correlation result indicates that different values of technical replicates presented similar alpha diversity patterns when different samples were included.

To evaluate whether the chosen sequence clustering method influences microbial diversity estimation, OTU-based and ASV-based clustering methods were assessed for their agreement regarding alpha and beta diversity pattern presentation. Linear regressions were applied to show the relationships between the OTU-based and ASV-based alpha diversity indices. The strong linear correlation between the OTU-based and ASV-based alpha diversity indices means that similar alpha diversity patterns were revealed by the two chosen clustering methods. A Procrustes analysis based on a principal coordinates analysis was used to test the agreement of the OTU-based and ASV-based distribution patterns, with the statistical significance being assessed via a Monte Carlo analysis and the M^2^ value being used to quantify the goodness-of-fit (38, 42).

All statistical analyses as well as the figure production were carried out using the R Project (version 3.6.1, https://www.r-project.org/), unless otherwise noted.

### Data availability.

All of the raw data were submitted to the NCBI Sequence Read Archive (accession code PRJNA845826).
