# Supplementary material for: Evaluating the Reproducibility of Amplicon Sequencing Data Derived from Deep-Sea Cold Seep Sediment-Associated Microbiota
Source: Microbiol Spectr. 2023 Apr 19;11(3):e04048-22. doi: 10.1128/spectrum.04048-22 (PMC10269476; doi:10.1128/spectrum.04048-22)
Supplement: Supplemental file 1 — Supplemental material. Download spectrum.04048-22-s0001.pdf, PDF file, 0.5 MB [file spectrum.04048-22-s0001.pdf]

Table S1 | The information of 344 sediment samples. Samples with two technical replicates were in bold. NCBI_id, sample names used for NCBI submission. The first four letters of each sample name indicate the station where it was collected. Depth, the distance relative to the top of sediment core.

NCBI_id	Depth	NCBI_id	Depth	NCBI_id	Depth
**ROV3S6A**	**6 cm**	ROV2S100D	100 cm	ROV4S160A	160 cm
**ROV3S6B**	**6 cm**	ROV2S120A	120 cm	ROV4S160B	160 cm
**ROV3S23A**	**23 cm**	ROV2S120B	120 cm	ROV4S160D	160 cm
**ROV3S23B**	**23 cm**	ROV2S120D	120 cm	ROV4S200A	200 cm
**ROV3S25A**	**25 cm**	ROV2S140A	140 cm	ROV4S200B	200 cm
**ROV3S25B**	**25 cm**	ROV2S140B	140 cm	ROV4S200D	200 cm
**ROV3S70A**	**70 cm**	ROV2S140D	140 cm	ROV4S250A	250 cm
**ROV3S70B**	**70 cm**	ROV2S160A	160 cm	ROV4S250B	250 cm
**ROV3S90A**	**90 cm**	ROV2S160B	160 cm	ROV4S250D	250 cm
**ROV3S90B**	**90 cm**	ROV2S160D	160 cm	ROV4S300A	300 cm
**ROV2S350A**	**350 cm**	ROV2S200A	200 cm	ROV4S300B	300 cm
**ROV2S350B**	**350 cm**	ROV2S200B	200 cm	ROV4S300D	300 cm
**ROV3S350A**	**350 cm**	ROV2S200D	200 cm	ROV4S350A	350 cm
**ROV3S350B**	**350 cm**	ROV2S250A	250 cm	ROV4S350B	350 cm
**ROV2S500B**	**500 cm**	ROV2S250B	250 cm	ROV4S350D	350 cm
**ROV2S500D**	**500 cm**	ROV2S250D	250 cm	ROV4S400A	400 cm
**ROV1S650A**	**650 cm**	ROV2S300A	300 cm	ROV4S400B	400 cm
**ROV1S650D**	**650 cm**	ROV2S300B	300 cm	ROV4S400D	400 cm
**ROV1S700A**	**700 cm**	ROV2S300D	300 cm	ROV4S450A	450 cm
**ROV1S700B**	**700 cm**	ROV2S400A	400 cm	ROV4S450B	450 cm
ROV1S5A	5 cm	ROV2S400B	400 cm	ROV4S450D	450 cm
ROV1S5B	5 cm	ROV2S400D	400 cm	ROV4S500A	500 cm
ROV1S5C	5 cm	ROV2S450A	450 cm	ROV4S500B	500 cm
ROV1S10A	10 cm	ROV2S450B	450 cm	ROV4S500D	500 cm
ROV1S10B	10 cm	ROV2S450D	450 cm	ROV4S550A	550 cm
ROV1S10D	10 cm	ROV2S550A	550 cm	ROV4S550B	550 cm
ROV1S15A	15 cm	ROV2S550B	550 cm	ROV4S550D	550 cm
ROV1S15B	15 cm	ROV2S550D	550 cm	ROV4S600A	600 cm
ROV1S15D	15 cm	ROV2S600A	600 cm	ROV4S600B	600 cm
ROV1S20A	20 cm	ROV2S600B	600 cm	ROV4S600D	600 cm
ROV1S20B	20 cm	ROV2S600	600 cm	ROV4S650A	650 cm
ROV1S20D	20 cm	ROV2S650A	650 cm	ROV4S650B	650 cm

ROV1S30A	30 cm	ROV2S650B	650 cm	ROV4S650D	650 cm
ROV1S30B	30 cm	ROV2S650D	650 cm	ROV4S700A	700 cm
ROV1S30D	30 cm	ROV3S10A	10 cm	ROV4S700B	700 cm
ROV1S40A	40 cm	ROV3S10B	10 cm	ROV4S700D	700 cm
ROV1S40B	40 cm	ROV3S10D	10 cm	ROV4S750A	750 cm
ROV1S40D	40 cm	ROV3S15A	15 cm	ROV4S750B	750 cm
ROV1S50A	50 cm	ROV3S15B	15 cm	ROV4S750	750 cm
ROV1S50B	50 cm	ROV3S15D	15 cm	ROV4S800A	800 cm
ROV1S50D	50 cm	ROV3S20A	20 cm	ROV4S800B	800 cm
ROV1S60A	60 cm	ROV3S20B	20 cm	ROV4S800D	800 cm
ROV1S60B	60 cm	ROV3S20D	20 cm	ROV5S5A	5 cm
ROV1S60D	60 cm	ROV3S30A	30 cm	ROV5S5B	5 cm
ROV1S80A	80 cm	ROV3S30B	30 cm	ROV5S5D	5 cm
ROV1S80B	80 cm	ROV3S30D	30 cm	ROV5S10A	10 cm
ROV1S80D	80 cm	ROV3S40A	40 cm	ROV5S10B	10 cm
ROV1S100A	100 cm	ROV3S40D	40 cm	ROV5S10D	10 cm
ROV1S100B	100 cm	ROV3S40	40 cm	ROV5S15A	15 cm
ROV1S100D	100 cm	ROV3S50A	50 cm	ROV5S15B	15 cm
ROV1S120AB	120 cm	ROV3S50B	50 cm	ROV5S15D	15 cm
ROV1S120B	120 cm	ROV3S50D	50 cm	ROV5S20A	20 cm
ROV1S120	120 cm	ROV3S60A	60 cm	ROV5S20B	20 cm
ROV1S140A	140 cm	ROV3S60B	60 cm	ROV5S20D	20 cm
ROV1S140B	140 cm	ROV3S60D	60 cm	ROV5S30A	30 cm
ROV1S140D	140 cm	ROV3S80A	80 cm	ROV5S30B	30 cm
ROV1S160A	160 cm	ROV3S80B	80 cm	ROV5S30D	30 cm
ROV1S160B	160 cm	ROV3S80D	80 cm	ROV5S40A	40 cm
ROV1S160D	160 cm	ROV3S150A	150 cm	ROV5S40B	40 cm
ROV1S200A	200 cm	ROV3S150B	150 cm	ROV5S40D	40 cm
ROV1S200B	200 cm	ROV3S150D	150 cm	ROV5S50A	50 cm
ROV1S200D	200 cm	ROV3S250A	250 cm	ROV5S50B	50 cm
ROV1S250A	250 cm	ROV3S250B	250 cm	ROV5S50D	50 cm
ROV1S250B	250 cm	ROV3S250D	250 cm	ROV5S60A	60 cm
ROV1S250D	250 cm	ROV3S450A	450 cm	ROV5S60B	60 cm
ROV1S300A	300 cm	ROV3S450B	450 cm	ROV5S60D	60 cm
ROV1S300B	300 cm	ROV3S450D	450 cm	ROV5S80A	80 cm
ROV1S300D	300 cm	ROV3S500A	500 cm	ROV5S80B	80 cm
ROV1S350A	350 cm	ROV3S500B	500 cm	ROV5S80D	80 cm
ROV1S350B	350 cm	ROV3S500D	500 cm	ROV5S100A	100 cm
ROV1S350D	350 cm	ROV3S550A	550 cm	ROV5S100B	100 cm
ROV1S400A	400 cm	ROV3S550B	550 cm	ROV5S100D	100 cm
ROV1S400D	400 cm	ROV3S550D	550 cm	ROV5S120A	120 cm

ROV1S400	400 cm	ROV3S600A	600 cm	ROV5S120B	120 cm
ROV1S450A	450 cm	ROV3S600B	600 cm	ROV5S120D	120 cm
ROV1S450B	450 cm	ROV3S600D	600 cm	ROV5S140A	140 cm
ROV1S450D	450 cm	ROV3S650A	650 cm	ROV5S140B	140 cm
ROV1S500A	500 cm	ROV3S650B	650 cm	ROV5S140D	140 cm
ROV1S500B	500 cm	ROV3S650D	650 cm	ROV5S160A	160 cm
ROV1S500D	500 cm	ROV3S700A	700 cm	ROV5S160B	160 cm
ROV1S550A	550 cm	ROV3S700B	700 cm	ROV5S160D	160 cm
ROV1S550B	550 cm	ROV3S700D	700 cm	ROV5S200A	200 cm
ROV1S550D	550 cm	ROV4S5A	5 cm	ROV5S200B	200 cm
ROV1S600A	600 cm	ROV4S5B	5 cm	ROV5S200D	200 cm
ROV1S600B	600 cm	ROV4S5D	5 cm	ROV5S250A	250 cm
ROV1S600D	600 cm	ROV4S10A	10 cm	ROV5S250B	250 cm
ROV2S5A	5 cm	ROV4S10B	10 cm	ROV5S250D	250 cm
ROV2S5B	5 cm	ROV4S10D	10 cm	ROV5S300A	300 cm
ROV2S5D	5 cm	ROV4S20B	20 cm	ROV5S300B	300 cm
ROV2S10A	10 cm	ROV4S20D	20 cm	ROV5S300D	300 cm
ROV2S10B	10 cm	ROV4S20	20 cm	ROV5S350A	350 cm
ROV2S10D	10 cm	ROV4S30A	30 cm	ROV5S350B	350 cm
ROV2S15A	15 cm	ROV4S30B	30 cm	ROV5S350D	350 cm
ROV2S15B	15 cm	ROV4S30D	30 cm	ROV5S400A	400 cm
ROV2S15D	15 cm	ROV4S40A	40 cm	ROV5S400B	400 cm
ROV2S20A	20 cm	ROV4S40B	40 cm	ROV5S400D	400 cm
ROV2S20B	20 cm	ROV4S40D	40 cm	ROV5S450A	450 cm
ROV2S20D	20 cm	ROV4S50A	50 cm	ROV5S450B	450 cm
ROV2S30A	30 cm	ROV4S50B	50 cm	ROV5S450D	450 cm
ROV2S30B	30 cm	ROV4S50D	50 cm	ROV5S500A	500 cm
ROV2S30D	30 cm	ROV4S60A	60 cm	ROV5S500B	500 cm
ROV2S40A	40 cm	ROV4S60B	60 cm	ROV5S500D	500 cm
ROV2S40B	40 cm	ROV4S60D	60 cm	ROV5S550A	550 cm
ROV2S40D	40 cm	ROV4S80A	80 cm	ROV5S550B	550 cm
ROV2S50A	50 cm	ROV4S80B	80 cm	ROV5S550	550 cm
ROV2S50B	50 cm	ROV4S80D	80 cm	ROV5S600A	600 cm
ROV2S50D	50 cm	ROV4S100A	100 cm	ROV5S600B	600 cm
ROV2S60A	60 cm	ROV4S100B	100 cm	ROV5S600D	600 cm
ROV2S60B	60 cm	ROV4S100D	100 cm	ROV5S650A	650 cm
ROV2S60D	60 cm	ROV4S120A	120 cm	ROV5S650B	650 cm
ROV2S80A	80 cm	ROV4S120B	120 cm	ROV5S650D	650 cm
ROV2S80B	80 cm	ROV4S120D	120 cm	ROV5S700A	700 cm
ROV2S80D	80 cm	ROV4S140A	140 cm	ROV5S700B	700 cm
ROV2S100A	100 cm	ROV4S140B	140 cm	ROV5S700D	700 cm

ROV2S100B	100 cm	ROV4S140D	140 cm

6

Table S2 | The number of reads before rarefaction for the 344 sediment samples. Samples with two technical replicates were in bold. NCBI_id, sample names used for NCBI submission. rOTU and rASV represent the read number kept after quality control (i.e. removing low-quality and non-bacterial reads) and before rarefaction for OTU- and ASV-based analyzing pipeline, respectively.

NCBI_id	rOTU	rASV	NCBI_id	rOTU	rASV	NCBI_id	rOTU	rASV
**ROV3S6A**	**66976**	**67711**	ROV2S100D	65021	67717	ROV4S160A	66176	68041
**ROV3S6B**	**66733**	**67580**	ROV2S120A	68078	68072	ROV4S160B	65105	67828
**ROV3S23A**	**66736**	**67722**	ROV2S120B	67088	67946	ROV4S160D	64707	66979
**ROV3S23B**	**66289**	**67484**	ROV2S120D	64877	67429	ROV4S200A	67731	64984
**ROV3S25A**	**66828**	**67545**	ROV2S140A	67294	67668	ROV4S200B	64796	67489
**ROV3S25B**	**66101**	**67924**	ROV2S140B	65219	67576	ROV4S200D	66016	67677
**ROV3S70A**	**66922**	**68171**	ROV2S140D	66246	67855	ROV4S250A	66738	67257
**ROV3S70B**	**65842**	**66716**	ROV2S160A	66706	67513	ROV4S250B	66247	68204
**ROV3S90A**	**66845**	**67594**	ROV2S160B	65425	68341	ROV4S250D	65025	67621
**ROV3S90B**	**66420**	**67304**	ROV2S160D	65104	67632	ROV4S300A	65820	66702
**ROV2S350A**	**65932**	**67644**	ROV2S200A	67487	68129	ROV4S300B	65487	67127
**ROV2S350B**	**66836**	**68063**	ROV2S200B	66267	67465	ROV4S300D	65528	67017
**ROV3S350A**	**66988**	**68158**	ROV2S200D	66399	68402	ROV4S350A	66304	68008
**ROV3S350B**	**66766**	**67516**	ROV2S250A	66250	67163	ROV4S350B	65748	66736
**ROV2S500B**	**66665**	**67957**	ROV2S250B	65330	67098	ROV4S350D	65992	66787
**ROV2S500D**	**64525**	**66424**	ROV2S250D	66124	68114	ROV4S400A	65016	67268
**ROV1S650A**	**57049**	**66952**	ROV2S300A	66876	67870	ROV4S400B	64177	65814
**ROV1S650D**	**64772**	**67478**	ROV2S300B	67100	68346	ROV4S400D	64517	66074
**ROV1S700A**	**65767**	**67233**	ROV2S300D	66460	67809	ROV4S450A	59014	59125
**ROV1S700B**	**66514**	**68058**	ROV2S400A	65942	67301	ROV4S450B	63806	64779
ROV1S5A	65814	67002	ROV2S400B	65428	67556	ROV4S450D	64830	66108
ROV1S5B	65153	65861	ROV2S400D	65860	67434	ROV4S500A	65643	65605
ROV1S5C	64776	66516	ROV2S450A	67621	68394	ROV4S500B	63967	62877
ROV1S10A	67110	68464	ROV2S450B	65238	67128	ROV4S500D	65413	65371
ROV1S10B	65286	67010	ROV2S450D	65327	68089	ROV4S550A	67378	68103
ROV1S10D	64697	67321	ROV2S550A	65906	67794	ROV4S550B	66375	68057
ROV1S15A	65390	66332	ROV2S550B	65814	67660	ROV4S550D	64430	67992
ROV1S15B	65809	67040	ROV2S550D	65533	64299	ROV4S600A	67521	67607
ROV1S15D	64961	66367	ROV2S600A	67178	67584	ROV4S600B	66329	67947
ROV1S20A	67022	67802	ROV2S600B	66088	68364	ROV4S600D	66011	67543

ROV1S20B	66365	67466	ROV2S600	64348	67720	ROV4S650A	66900	67555
ROV1S20D	65189	68079	ROV2S650A	62049	67245	ROV4S650B	65950	67602
ROV1S30A	66743	67483	ROV2S650B	65437	67468	ROV4S650D	64094	67737
ROV1S30B	67382	67893	ROV2S650D	64785	67520	ROV4S700A	65857	67826
ROV1S30D	65063	67682	ROV3S10A	66345	67367	ROV4S700B	65457	68359
ROV1S40A	66798	68085	ROV3S10B	65634	67194	ROV4S700D	65600	67974
ROV1S40B	65955	67568	ROV3S10D	66769	67878	ROV4S750A	65955	67321
ROV1S40D	64744	67668	ROV3S15A	66906	66792	ROV4S750B	65380	67938
ROV1S50A	66889	67297	ROV3S15B	66875	68107	ROV4S750	63626	67507
ROV1S50B	65765	67464	ROV3S15D	66455	67766	ROV4S800A	65535	67996
ROV1S50D	66317	68217	ROV3S20A	66157	67519	ROV4S800B	64650	67349
ROV1S60A	67539	67993	ROV3S20B	66168	67558	ROV4S800D	66422	68285
ROV1S60B	67007	67954	ROV3S20D	65351	67097	ROV5S5A	65174	66949
ROV1S60D	65342	66959	ROV3S30A	67311	68406	ROV5S5B	66067	67445
ROV1S80A	66634	67800	ROV3S30B	65192	67290	ROV5S5D	65598	67946
ROV1S80B	65965	67360	ROV3S30D	64570	68118	ROV5S10A	66340	67757
ROV1S80D	64781	67356	ROV3S40A	66681	68170	ROV5S10B	65574	67746
ROV1S100A	67712	68637	ROV3S40D	64876	67007	ROV5S10D	64961	67587
ROV1S100B	65301	67317	ROV3S40	66500	67910	ROV5S15A	66796	67305
ROV1S100D	66486	68155	ROV3S50A	66221	66557	ROV5S15B	67230	68445
ROV1S120AB	65747	67915	ROV3S50B	65754	67763	ROV5S15D	65077	66923
ROV1S120B	64812	67409	ROV3S50D	64087	67081	ROV5S20A	67938	68494
ROV1S120	66656	66862	ROV3S60A	65968	67951	ROV5S20B	64322	67181
ROV1S140A	66628	67137	ROV3S60B	67843	68461	ROV5S20D	64475	67713
ROV1S140B	66615	67749	ROV3S60D	65375	68075	ROV5S30A	66321	66931
ROV1S140D	65492	67123	ROV3S80A	66197	67391	ROV5S30B	66450	67643
ROV1S160A	66910	67707	ROV3S80B	66156	67759	ROV5S30D	64069	66704
ROV1S160B	65482	68410	ROV3S80D	64733	67573	ROV5S40A	66924	67743
ROV1S160D	64983	67851	ROV3S150A	67126	68042	ROV5S40B	64982	67558
ROV1S200A	65214	67440	ROV3S150B	66719	67835	ROV5S40D	65476	67822
ROV1S200B	65784	67951	ROV3S150D	64510	67314	ROV5S50A	65570	67467
ROV1S200D	64348	67717	ROV3S250A	66660	67516	ROV5S50B	65319	67864
ROV1S250A	66038	67312	ROV3S250B	64958	67519	ROV5S50D	66549	67624
ROV1S250B	65942	67077	ROV3S250D	65947	68507	ROV5S60A	66928	68134
ROV1S250D	66733	68505	ROV3S450A	65354	67287	ROV5S60B	64448	67442
ROV1S300A	65796	67323	ROV3S450B	66609	68353	ROV5S60D	65313	68497
ROV1S300B	65950	68403	ROV3S450D	65521	67115	ROV5S80A	65834	67054
ROV1S300D	64843	67138	ROV3S500A	66929	67937	ROV5S80B	65349	67251
ROV1S350A	66358	67437	ROV3S500B	65431	67740	ROV5S80D	65934	68166
ROV1S350B	67090	68012	ROV3S500D	64072	67603	ROV5S100A	66700	67840
ROV1S350D	66162	67564	ROV3S550A	66331	67438	ROV5S100B	65505	67770

ROV1S400A	66764	67914	ROV3S550B	64756	67519	ROV5S100D	64514	67770
ROV1S400D	65273	67488	ROV3S550D	64919	68228	ROV5S120A	66162	67302
ROV1S400	65940	67788	ROV3S600A	67779	68532	ROV5S120B	66967	68605
ROV1S450A	66800	67746	ROV3S600B	65269	67220	ROV5S120D	64663	67067
ROV1S450B	66180	67347	ROV3S600D	64333	67263	ROV5S140A	66081	67616
ROV1S450D	65563	67998	ROV3S650A	66109	67134	ROV5S140B	66646	67716
ROV1S500A	66564	67356	ROV3S650B	66184	67874	ROV5S140D	65640	67282
ROV1S500B	67589	68228	ROV3S650D	63403	67387	ROV5S160A	66226	67590
ROV1S500D	66528	64752	ROV3S700A	67958	66896	ROV5S160B	67280	68505
ROV1S550A	66275	67219	ROV3S700B	65273	67693	ROV5S160D	65490	67240
ROV1S550B	66643	67889	ROV3S700D	63786	68062	ROV5S200A	67313	68051
ROV1S550D	65355	67303	ROV4S5A	66857	67524	ROV5S200B	67212	67780
ROV1S600A	66842	68057	ROV4S5B	66156	66717	ROV5S200D	59066	60128
ROV1S600B	67378	68166	ROV4S5D	65845	65539	ROV5S250A	66782	67682
ROV1S600D	66986	68222	ROV4S10A	67396	67707	ROV5S250B	66653	67479
ROV2S5A	66328	66830	ROV4S10B	66005	67476	ROV5S250D	59648	60970
ROV2S5B	66341	67207	ROV4S10D	66342	68589	ROV5S300A	66192	66611
ROV2S5D	66711	67983	ROV4S20B	66025	67525	ROV5S300B	65508	66735
ROV2S10A	66531	66861	ROV4S20D	64724	67160	ROV5S300D	63165	63923
ROV2S10B	67178	68050	ROV4S20	66495	67261	ROV5S350A	66352	67630
ROV2S10D	64717	67278	ROV4S30A	66133	67666	ROV5S350B	66888	67554
ROV2S15A	66732	67640	ROV4S30B	66608	67930	ROV5S350D	66148	66960
ROV2S15B	64636	66574	ROV4S30D	65829	67792	ROV5S400A	67290	67770
ROV2S15D	65546	67483	ROV4S40A	66972	67658	ROV5S400B	66707	67426
ROV2S20A	67212	67883	ROV4S40B	66604	67824	ROV5S400D	66121	67018
ROV2S20B	65991	67602	ROV4S40D	64393	67356	ROV5S450A	67158	68246
ROV2S20D	65569	68147	ROV4S50A	67853	68549	ROV5S450B	66813	67252
ROV2S30A	67518	67535	ROV4S50B	64735	67412	ROV5S450D	67032	67826
ROV2S30B	66568	67308	ROV4S50D	63552	67651	ROV5S500A	65761	66865
ROV2S30D	65726	67773	ROV4S60A	66285	67123	ROV5S500B	66819	66587
ROV2S40A	66352	67295	ROV4S60B	66016	68190	ROV5S500D	59629	60688
ROV2S40B	65993	67880	ROV4S60D	64993	67572	ROV5S550A	66862	67709
ROV2S40D	65685	67482	ROV4S80A	67830	67521	ROV5S550B	67062	67829
ROV2S50A	66626	68012	ROV4S80B	65733	67125	ROV5S550	66488	67259
ROV2S50B	67160	67679	ROV4S80D	65741	67578	ROV5S600A	66611	67810
ROV2S50D	66919	68226	ROV4S100A	66485	66762	ROV5S600B	67596	68552
ROV2S60A	66012	66966	ROV4S100B	66248	67806	ROV5S600D	66668	67861
ROV2S60B	66474	68051	ROV4S100D	64332	67964	ROV5S650A	67749	68232
ROV2S60D	65617	68103	ROV4S120A	66643	67496	ROV5S650B	66651	67115
ROV2S80A	67312	67753	ROV4S120B	65158	67995	ROV5S650D	66123	66833
ROV2S80B	66267	67877	ROV4S120D	64132	67568	ROV5S700A	66722	67669

ROV2S80D	66982	68626	ROV4S140A	66689	66875	ROV5S700B	67186	67724
ROV2S100A	66811	68374	ROV4S140B	65610	67503	ROV5S700D	66303	67449
ROV2S100B	65555	67139	ROV4S140D	64063	67340			

Table S3 | The number of OTUs produced when the sequences were not rarefied and rarefied for the 344 sediment samples. Samples with two technical replicates were in bold. NCBI_id, sample names used for NCBI submission. Before and After represent the number of OTUs produced when the sequences were not rarefied and rarefied, respectively.

NCBI_id	Before	After	NCBI_id	Before	After	NCBI_id	Before	After
**ROV3S6A**	**526**	**504**	ROV2S100D	110	106	ROV4S160A	159	154
**ROV3S6B**	**495**	**474**	ROV2S120A	192	154	ROV4S160B	263	250
**ROV3S23A**	**279**	**273**	ROV2S120B	170	164	ROV4S160D	138	137
**ROV3S23B**	**187**	**180**	ROV2S120D	176	168	ROV4S200A	244	232
**ROV3S25A**	**1000**	**932**	ROV2S140A	102	89	ROV4S200B	343	329
**ROV3S25B**	**732**	**711**	ROV2S140B	85	80	ROV4S200D	167	160
**ROV3S70A**	**88**	**84**	ROV2S140D	74	70	ROV4S250A	364	354
**ROV3S70B**	**283**	**257**	ROV2S160A	125	123	ROV4S250B	226	223
**ROV3S90A**	**157**	**145**	ROV2S160B	108	99	ROV4S250D	144	137
**ROV3S90B**	**124**	**108**	ROV2S160D	134	122	ROV4S300A	1115	1064
**ROV2S350A**	**928**	**881**	ROV2S200A	137	128	ROV4S300B	1248	1167
**ROV2S350B**	**463**	**438**	ROV2S200B	110	108	ROV4S300D	966	901
**ROV3S350A**	**1117**	**1031**	ROV2S200D	115	110	ROV4S350A	1155	1092
**ROV3S350B**	**396**	**375**	ROV2S250A	260	252	ROV4S350B	1317	1220
**ROV2S500B**	**168**	**159**	ROV2S250B	376	353	ROV4S350D	1332	1226
**ROV2S500D**	**139**	**133**	ROV2S250D	272	263	ROV4S400A	1195	1126
**ROV1S650A**	**209**	**208**	ROV2S300A	259	249	ROV4S400B	1563	1433
**ROV1S650D**	**123**	**120**	ROV2S300B	230	217	ROV4S400D	1612	1481
**ROV1S700A**	**79**	**75**	ROV2S300D	389	359	ROV4S450A	2340	2014
**ROV1S700B**	**75**	**69**	ROV2S400A	225	217	ROV4S450B	2420	2060
ROV1S5A	1127	1088	ROV2S400B	249	233	ROV4S450D	2003	1798
ROV1S5B	1970	1870	ROV2S400D	190	180	ROV4S500A	1798	1634
ROV1S5C	1274	1215	ROV2S450A	81	74	ROV4S500B	2082	1818
ROV1S10A	356	345	ROV2S450B	107	99	ROV4S500D	1926	1734
ROV1S10B	532	524	ROV2S450D	96	93	ROV4S550A	260	240
ROV1S10D	336	328	ROV2S550A	102	94	ROV4S550B	327	324
ROV1S15A	1185	1135	ROV2S550B	84	81	ROV4S550D	214	205
ROV1S15B	1282	1232	ROV2S550D	121	117	ROV4S600A	338	324
ROV1S15D	1284	1228	ROV2S600A	58	51	ROV4S600B	505	477
ROV1S20A	184	179	ROV2S600B	63	58	ROV4S600D	180	169

ROV1S20B	196	182	ROV2S600	60	56	ROV4S650A	147	132
ROV1S20D	183	177	ROV2S650A	247	236	ROV4S650B	277	268
ROV1S30A	135	131	ROV2S650B	65	64	ROV4S650D	151	144
ROV1S30B	526	500	ROV2S650D	77	70	ROV4S700A	156	145
ROV1S30D	210	203	ROV3S10A	490	470	ROV4S700B	141	133
ROV1S40A	137	132	ROV3S10B	808	763	ROV4S700D	77	73
ROV1S40B	112	107	ROV3S10D	804	771	ROV4S750A	208	195
ROV1S40D	166	162	ROV3S15A	174	165	ROV4S750B	76	70
ROV1S50A	136	132	ROV3S15B	692	648	ROV4S750	71	63
ROV1S50B	107	105	ROV3S15D	342	328	ROV4S800A	245	232
ROV1S50D	136	130	ROV3S20A	479	459	ROV4S800B	107	103
ROV1S60A	92	90	ROV3S20B	599	568	ROV4S800D	69	64
ROV1S60B	78	76	ROV3S20D	657	625	ROV5S5A	524	507
ROV1S60D	215	204	ROV3S30A	126	123	ROV5S5B	533	506
ROV1S80A	105	92	ROV3S30B	257	248	ROV5S5D	349	334
ROV1S80B	121	118	ROV3S30D	91	90	ROV5S10A	425	412
ROV1S80D	157	148	ROV3S40A	291	282	ROV5S10B	592	571
ROV1S100A	159	156	ROV3S40D	427	411	ROV5S10D	303	290
ROV1S100B	122	115	ROV3S40	448	432	ROV5S15A	437	412
ROV1S100D	133	128	ROV3S50A	137	128	ROV5S15B	776	748
ROV1S120AB	326	315	ROV3S50B	87	80	ROV5S15D	659	621
ROV1S120B	323	308	ROV3S50D	81	78	ROV5S20A	119	113
ROV1S120	275	266	ROV3S60A	115	110	ROV5S20B	159	148
ROV1S140A	118	116	ROV3S60B	129	118	ROV5S20D	129	124
ROV1S140B	69	65	ROV3S60D	91	85	ROV5S30A	246	220
ROV1S140D	123	117	ROV3S80A	190	183	ROV5S30B	491	472
ROV1S160A	261	250	ROV3S80B	350	339	ROV5S30D	250	243
ROV1S160B	168	160	ROV3S80D	117	108	ROV5S40A	225	220
ROV1S160D	190	174	ROV3S150A	92	87	ROV5S40B	220	214
ROV1S200A	115	93	ROV3S150B	110	96	ROV5S40D	132	130
ROV1S200B	106	100	ROV3S150D	104	98	ROV5S50A	114	104
ROV1S200D	147	134	ROV3S250A	129	112	ROV5S50B	145	139
ROV1S250A	723	675	ROV3S250B	173	161	ROV5S50D	247	192
ROV1S250B	240	205	ROV3S250D	99	89	ROV5S60A	147	136
ROV1S250D	131	111	ROV3S450A	95	89	ROV5S60B	221	211
ROV1S300A	204	195	ROV3S450B	205	191	ROV5S60D	137	128
ROV1S300B	256	235	ROV3S450D	96	91	ROV5S80A	92	86
ROV1S300D	195	190	ROV3S500A	209	192	ROV5S80B	117	111
ROV1S350A	1175	1086	ROV3S500B	148	142	ROV5S80D	94	88
ROV1S350B	176	155	ROV3S500D	251	234	ROV5S100A	115	109
ROV1S350D	307	297	ROV3S550A	135	131	ROV5S100B	447	431

ROV1S400A	103	94	ROV3S550B	113	108	ROV5S100D	196	185
ROV1S400D	95	85	ROV3S550D	106	105	ROV5S120A	168	152
ROV1S400	102	93	ROV3S600A	110	99	ROV5S120B	300	290
ROV1S450A	162	158	ROV3S600B	88	85	ROV5S120D	110	106
ROV1S450B	245	236	ROV3S600D	101	98	ROV5S140A	386	371
ROV1S450D	140	127	ROV3S650A	144	137	ROV5S140B	365	351
ROV1S500A	77	73	ROV3S650B	67	62	ROV5S140D	344	332
ROV1S500B	68	63	ROV3S650D	66	63	ROV5S160A	320	306
ROV1S500D	195	190	ROV3S700A	69	66	ROV5S160B	339	324
ROV1S550A	95	92	ROV3S700B	57	57	ROV5S160D	1064	997
ROV1S550B	75	67	ROV3S700D	84	76	ROV5S200A	617	582
ROV1S550D	68	67	ROV4S5A	699	668	ROV5S200B	343	311
ROV1S600A	70	60	ROV4S5B	1448	1375	ROV5S200D	784	751
ROV1S600B	63	62	ROV4S5D	2134	2022	ROV5S250A	367	354
ROV1S600D	76	73	ROV4S10A	429	413	ROV5S250B	565	527
ROV2S5A	926	888	ROV4S10B	660	634	ROV5S250D	1058	994
ROV2S5B	1169	1107	ROV4S10D	421	403	ROV5S300A	2269	2061
ROV2S5D	981	939	ROV4S20B	478	456	ROV5S300B	1115	1046
ROV2S10A	328	314	ROV4S20D	172	161	ROV5S300D	1369	1263
ROV2S10B	332	316	ROV4S20	369	358	ROV5S350A	1108	1040
ROV2S10D	150	137	ROV4S30A	333	321	ROV5S350B	1065	997
ROV2S15A	198	178	ROV4S30B	349	342	ROV5S350D	1273	1194
ROV2S15B	121	112	ROV4S30D	362	344	ROV5S400A	397	376
ROV2S15D	286	272	ROV4S40A	195	189	ROV5S400B	767	715
ROV2S20A	113	105	ROV4S40B	215	204	ROV5S400D	837	786
ROV2S20B	98	94	ROV4S40D	167	156	ROV5S450A	702	649
ROV2S20D	116	112	ROV4S50A	122	118	ROV5S450B	366	341
ROV2S30A	116	105	ROV4S50B	171	167	ROV5S450D	608	584
ROV2S30B	87	86	ROV4S50D	96	95	ROV5S500A	459	438
ROV2S30D	119	109	ROV4S60A	151	145	ROV5S500B	563	537
ROV2S40A	418	405	ROV4S60B	200	192	ROV5S500D	604	573
ROV2S40B	137	133	ROV4S60D	167	164	ROV5S550A	716	664
ROV2S40D	126	118	ROV4S80A	149	140	ROV5S550B	326	310
ROV2S50A	224	215	ROV4S80B	157	151	ROV5S550	720	681
ROV2S50B	159	158	ROV4S80D	130	123	ROV5S600A	439	421
ROV2S50D	97	91	ROV4S100A	190	176	ROV5S600B	278	252
ROV2S60A	114	103	ROV4S100B	190	186	ROV5S600D	774	717
ROV2S60B	136	133	ROV4S100D	156	151	ROV5S650A	381	358
ROV2S60D	112	107	ROV4S120A	144	137	ROV5S650B	367	343
ROV2S80A	94	87	ROV4S120B	168	160	ROV5S650D	829	775
ROV2S80B	141	126	ROV4S120D	208	170	ROV5S700A	712	673

ROV2S80D	99	89	ROV4S140A	199	192	ROV5S700B	517	495
ROV2S100A	97	92	ROV4S140B	299	284	ROV5S700D	806	756
ROV2S100B	105	95	ROV4S140D	205	198			

Table S4 | Statistical summary of the values of the read counts in Table S2 and the OTU numbers in Table S3. rOTU and rASV represent the read number kept after quality control (i.e. removing low-quality and non-bacterial reads) and before rarefaction for OTU- and ASV-based analyzing pipeline, respectively. Before and After represent the number of OTUs produced when the sequences were not rarefied and rarefied, respectively. Min, minimum; Max, maximum; SD, standard deviation; CV, coefficient of variation.

	rOTU	rASV	Before	After
Min	57049	59125	57	51
Mean	65895	67436	373	350
Max	68078	68637	2420	2061
SD	1295	1046	428	393
%CV	1.96	1.55	115	112

Table S5 | OTU- and ASV-based overlap between two and among three technical replicates. Two and Three represent dataset with two and three technical replicates, respectively. Min, minimum; Max, maximum; SD, standard deviation. Unique, unique to one technical replicate; Shared.2, shared by two technical replicates; Shared.3, shared by three technical replicates.

Categories			Occurrence				Abundance			
			Min	Mean	Max	SD	Min	Mean	Max	SD
Two	OTU	Unique	52.82	64.02	81.37	8.75	4.25	15.12	33.23	8.80
		Shared.2	18.63	35.98	47.18	8.75	66.77	84.88	95.75	8.80
	ASV	Unique	54.77	67.26	83.00	8.60	6.40	22.36	41.02	12.07
		Shared.2	17.00	32.74	45.23	8.60	58.98	77.64	93.60	12.07
Three	OTU	Unique	26.75	49.86	67.59	7.98	0.98	9.54	21.74	4.31
		Shared.2	12.97	23.13	33.00	3.19	2.85	7.31	14.29	3.11
		Shared.3	13.28	27.02	50.15	6.90	67.18	83.16	94.19	6.44
	ASV	Unique	24.03	55.92	73.73	8.88	2.04	14.13	30.13	6.41
		Shared.2	13.17	20.09	31.63	3.58	3.55	8.29	17.69	3.45
		Shared.3	8.92	23.99	48.84	6.97	58.00	77.58	91.36	8.94

Table S6 | Statistical summary of the values of percent relative range (%) for alpha indices generated with and without sequence rarefaction. The percent relative range (PRR), the percentage ratio of the range to the average value. For each of the 344 samples, a PRR value was calculated. The larger PRR value means the more variability between values of alpha indices calculated with and without sequence rarefaction. OTU and ASV represent the sequence clustering method of dataset. Min, minimum; Max, maximum; SD, standard deviation.

	OTU		ASV	
	Richness	Shannon	Richness	Shannon
Min	0	0	0	0
Mean	5.6	0.3	5.7	0.3
Max	22.3	1.8	23.9	1.3

Table S7 | OTU- and ASV-based overlap between two and among three technical replicates after filtering ASVs/OTUs with sequences <100. Two and Three represent dataset with two and three technical replicates, respectively. Min, minimum; Max, maximum; SD, standard deviation. Unique, unique to one technical replicate; Shared.2, shared by two technical replicates; Shared.3, shared by three technical replicates.

Categories			Occurrence				Abundance			
			Min	Mean	Max	SD	Min	Mean	Max	SD
Two	OTU	Unique	39.01	60.61	83.61	14.32	3.61	10.54	31.20	8.12
		Shared.2	16.39	39.39	60.99	14.32	68.80	89.46	96.39	8.12
	ASV	Unique	41.46	62.44	83.49	12.94	5.23	14.47	436.03	9.14
		Shared.2	16.51	37.56	58.54	12.94	63.97	85.53	94.75	9.14
Three	OTU	Unique	29.73	65.12	79.55	9.63	0.87	8.25	19.65	3.69
		Shared.2	9.35	18.97	34.02	4.05	1.55	7.36	17.04	3.08
		Shared.3	6.32	15.91	51.72	7.07	68.05	84.39	97.59	5.75
	ASV	Unique	29.70	67.38	81.12	9.61	1.49	11.57	25.34	4.74
		Shared.2	9.97	17.41	30.18	4.19	2.90	8.82	20.41	3.26
		Shared.3	5.16	15.22	48.15	6.56	64.04	79.61	95.62	6.70

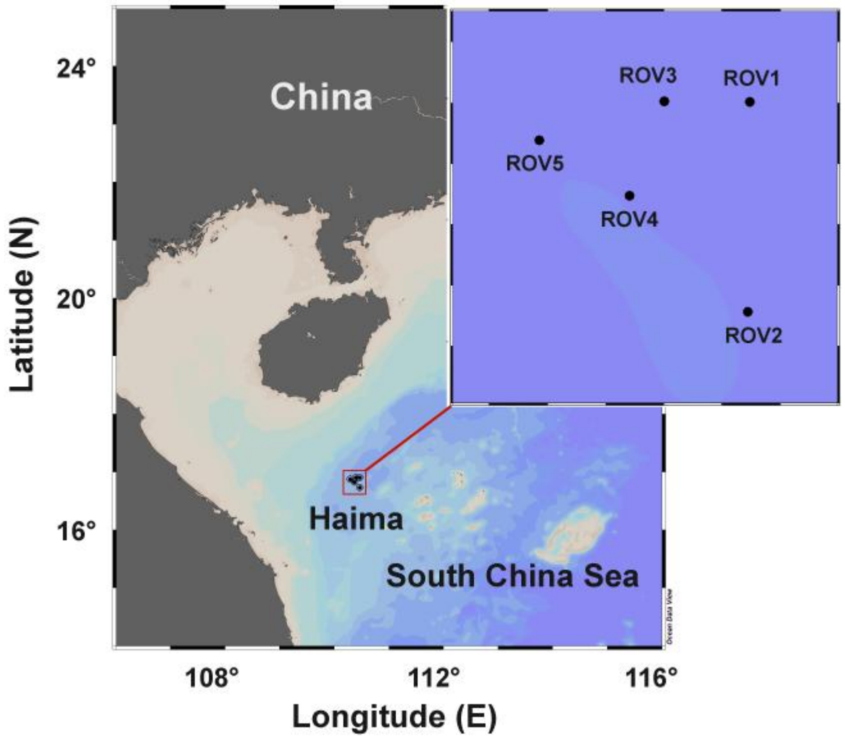

Figure S1 | Sampling locations in the Northern South China Sea. The map with sampling locations was constructed with Ocean Data View version 4.7.10.

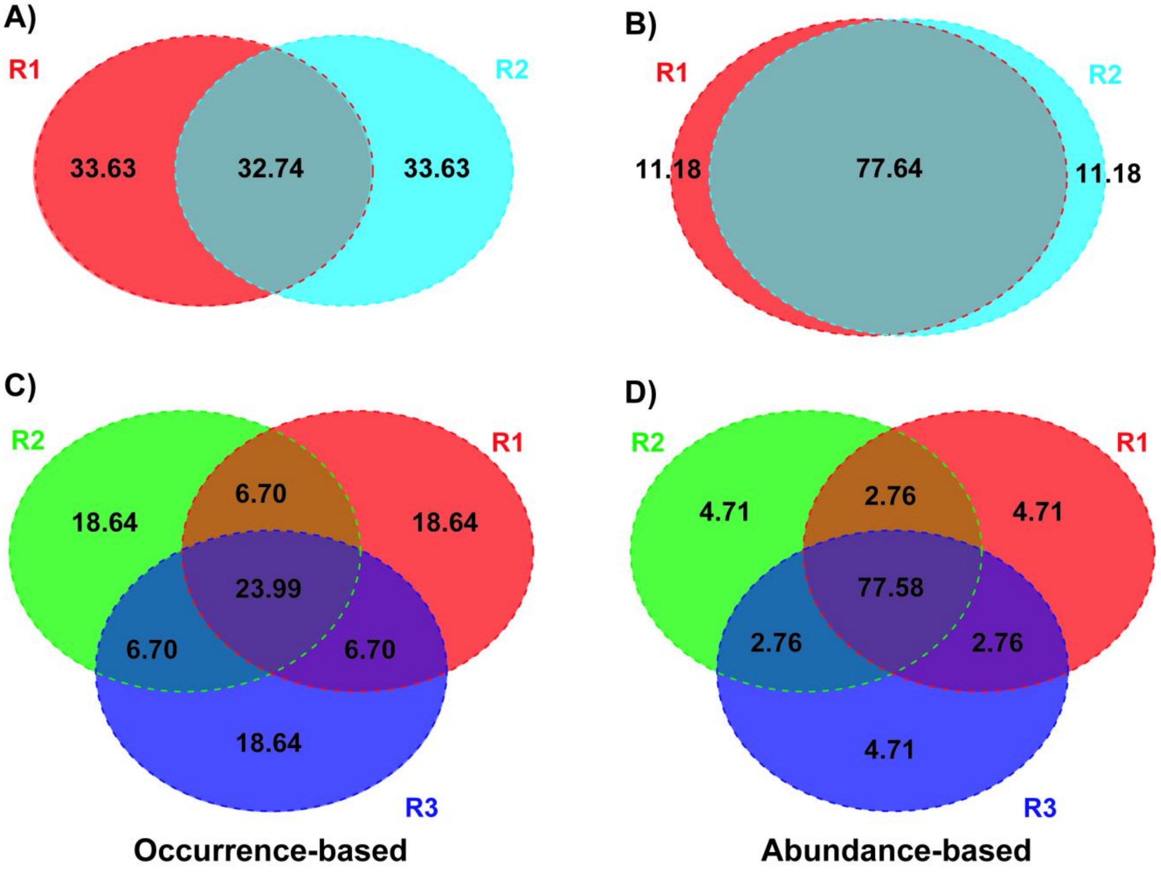

~~**Figure S2 | The average ASV-based overlap between two (A) and B)) and among three (C) and D)) technical replicates.** In **A)** and **C)**, only occurrence (presence or absence) of ASVs was considered, while in **B)** and **D)**, the sequence abundances of ASVs were considered. R1, R2 and R3 represent one of the technical replicate, respectively. For each sample, a value of ASV-based overlap between two or among three technical replicates was calculated. The Venn diagram shows the average.~~

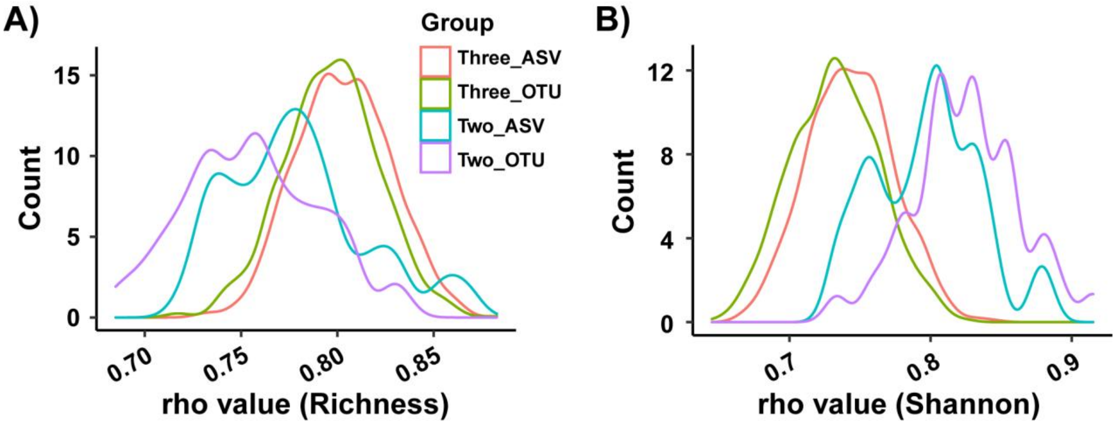

Figure ~~S3~~ S2 | Distributions of Spearman's rho values for A) Richness and B) Shannon indices among technical replicates. Random division and spearman correlation test were repeated 999 times. All *p* values of Spearman correlation tests were <0.05. Two and Three represent dataset with two and three technical replicates, respectively.

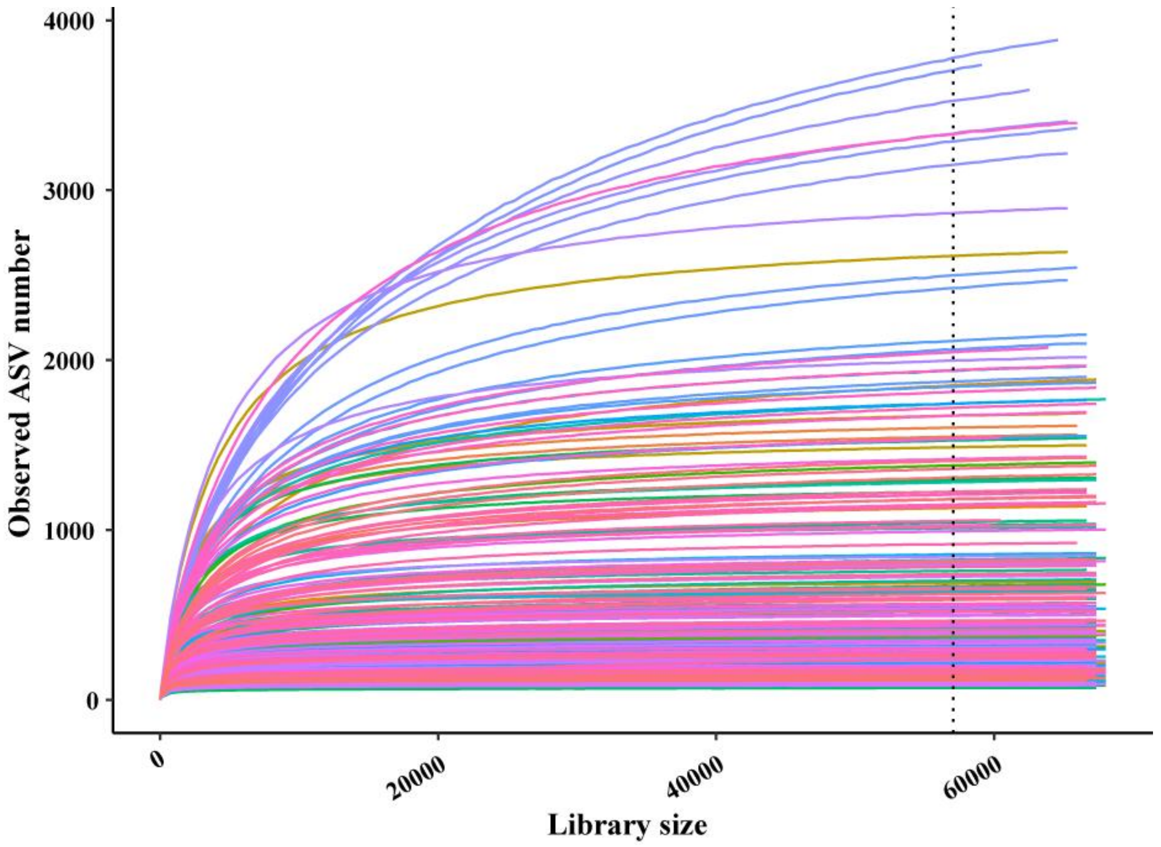

Figure ~~S4~~ S3 | Rarefaction curves showing the number of ASVs as a function of normalized library size for the 344 samples. Each sample was rarefied for different library sizes repeatedly (100 times) to generate a representative set of data for rarefaction curve construction. The vertical dotted black line represent the lowest sequence number (57047) rarefied in this study. Repeated rarefaction was achieved by using function rarefy_whole_rep() in R package "mirlyn" (version 1.3.0, https://github.com/escamero/mirlyn/).
